# Apnoeic oxygenation in pediatric anesthesia: better safe than sorry!

**DOI:** 10.1186/s12871-025-02995-2

**Published:** 2025-03-08

**Authors:** Davut Deniz Uzun, Felix Hezel, Stefan Mohr, Markus A. Weigand, Felix C. F. Schmitt

**Affiliations:** Medical Faculty Heidelberg, Department of Anesthesiology, Heidelberg University, Heidelberg, Germany

**Keywords:** Airway management, Apnoeic oxygenation, Pediatric anesthesia, Oxygen, Tracheal intubation

## Abstract

**Background:**

Children, especially neonates and infants, are at particularly high risk of hypoxemia during induction of anesthesia. The addition of nasal apnoeic oxygenation (ApOx) during tracheal intubation should prolong safe apnoea time without desaturation and reduce the risk of hypoxemia. Despite the recommendations in the relevant European guidelines, their implementation in pediatric anesthesia in Germany is not yet known.

**Methods:**

A survey was conducted in July and October 2024 via email to all registered members of the scientific working group on airway management, the scientific working group on pediatric anesthesia of the German Society of Anesthesiology and Intensive Care Medicine (DGAI) and hospitals of all levels in Germany. Participants were asked about their personal and institutional background and the use of ApOx in pediatric anesthesia in their institution.

**Results:**

Of the eight hundred participants invited, 304 anesthetists completed the survey (response rate 38%). In addition, 36 of 109 invited anesthetists from the scientific working group on pediatric anesthesia were interviewed as a separate expert group. 201 (66.1%) of the anesthetists surveyed in the general group stated that they worked regular in pediatric anesthesia (pediatric anesthesia expert group: 94.4%). 64.2% of the general respondents considered pediatric patients to be at an increased risk of reduced apnoea time. 46.7% of the general participants are of the opinion that pediatric patients should generally not receive ApOx during induction of anesthesia. If ApOx is performed, then most likely with a standard nasal cannula. ApOx was generally used in infants with an oxygen flow rate of ≤ 2 l/min or 0.2 l/kg bodyweight/min. A relevant proportion of anesthetists were unaware that current European guidelines recommend ApOx for neonates and infants (general participants: 62.5%, pediatric anesthesia expert group: 39%).

**Conclusions:**

Despite the recommendations in the guidelines, the use of ApOx does not appear to be standard practice at present. Furthermore, the surveyed physicians exhibited considerable uncertainty regarding ApOx. It is imperative that further improvements are made in the dissemination of the current guidelines with a view to enhancing patient safety during pediatric anesthesia.

**Supplementary Information:**

The online version contains supplementary material available at 10.1186/s12871-025-02995-2.

## Background

Desaturation during anesthesia induction is more prevalent, accelerated and severe in infants and children due to their unique physiology. The consequences of prolonged desaturation are potentially dramatic [1, 2]. In this context, the lethal triad of hypoxia, hypotension and anemia should be mentioned during pediatric anesthesia [3]. The prevalence of intraoperative hypoxemia in neonates and infants is high and correlates with the age. An incidence of over 10% has been documented in older children (> 8 years), while rates of over 50% have been observed in neonates [4]. A important goal during tracheal intubation is to successfully complete the procedure on the first attempt [5]. The incidence of a difficult airway condition in neonates and infants, is approximately 5.8% and is typically anticipated [3]. It is therefore imperative that every effort is made to prevent desaturation during the induction of anesthesia. Such adverse events have the potential to result in hypoxemia, cardiac arrest, or even death [2, 6–8]. A number of techniques may be employed in order to extend the safe apnoea time without desaturation. The following methods have been established as means of increasing the apnoea time without desaturation: These include preoxygenation, the administration of 100% fractional inspired oxygen (FIO2), positive end-expiratory pressure (PEEP) during preoxygenation, low-flow or high-flow apnoeic oxygenation (ApOx) during tracheal intubation, and the limitation of the number of attempts and the time required [2, 9]. In light of the aforementioned considerations, it is recommended that all available options be utilized to extend the safe apnoea time without desaturation in children.

**Fig. 1:**
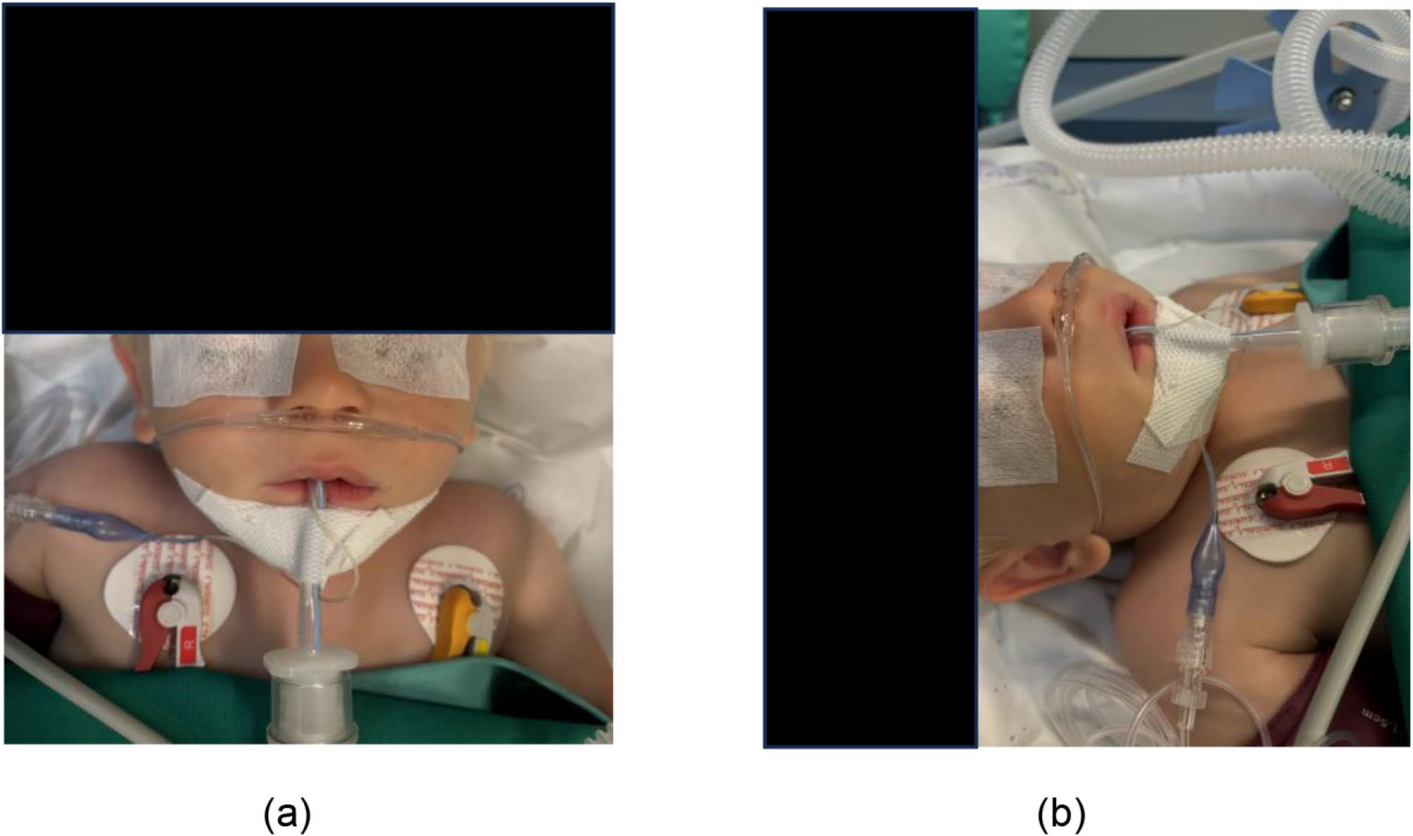
(**a**) and (**b**) show the low-flow nasal cannula as it was used for apnoeic oxygenation (ApOx) before endotracheal intubation. Note: The picture was taken after tracheal intubation

In view of the importance of this problem and its consequences, European guidelines for airway management in neonates and infants have been published in January 2024 (Epub 13/12/2023) [10]. The recommendations include the use of apnoeic oxygenation during tracheal intubation in newborns (1B), in infants, the use of apnoeic oxygenation should be based on the risk of hypoxemia in the patient and the experience of the provider (Clinical practice statement). The aim of these is to increase the safe apnoea time or the apnoea time without/before desaturation. The experts report that the optimal oxygen flow has yet to be determined [10]. The concept of apnoeic oxygenation is not a novel innovation; it has been known and described since the early 1900s [11]. From a technical perspective, there are a number of potential avenues for pursuing this method. The most cost-effective and ubiquitously available method is ApOx via a standard nasal cannula (Fig. 1). No additional devices or specialised machines are required for this procedure. There is growing evidence in the scientific literature that the use of ApOx is associated with several benefits for children [2, 12, 13]. A meta-analysis has been conducted that confirms the hypothesis that the use of ApOx during tracheal intubation in children significantly increases the success rate of the initial intubation attempt. Moreover, ApOx has been demonstrated to facilitate the establishment of stable physiological conditions by ensuring that oxygen saturation remains within the normal range [2, 14]. Earlier practice surveys also demonstrate a considerable degree of heterogeneity in the various measures and their application in the context of pediatric airway management and ventilation. This underscores the progress achieved in pediatric anesthesia and the challenges that persist [15, 16]. To the best of our knowledge, no real-world data is available for the use of ApOx in pediatric anesthesia in Germany. Therefore the objective of this study is to ascertain the frequency of ApOx utilisation and the methods employed in German anesthesia clinics.

## Methods

### Study design

In this multicenter explorative, web-based anonymous survey, anesthetists in various German hospitals at all levels of care were interviewed. Participants were able to access the survey via an anonymous (non-personalised) web-link. The survey was conducted over the course of one month, from 01. July to 31 July 2024 and in a second part from 01.October to 31.October 2024, for the pediatric anesthesia scientific working group of the German Society of Anesthesiology and Intensive Care Medicine (DGAI). The web-link was subsequently deactivated. After confirming the survey’s data protection regulations, they were able to start answering the questions on ApOx immediately. This survey was performed following institutional guidelines and the Declaration of Helsinki of 1975 in its most recent version. This study adheres to the standards set forth by the STROBE statement [17].

### Sampling strategy

The web-link to the survey was distributed to all registered members of the scientific airway management working group of the German Society of Anesthesiology and Intensive Care Medicine (DGAI) in July 2024. In a second part, in October 2024, the scientific pediatric anesthesia working group of the DGAI was surveyed. Additionally, university hospitals and other hospitals at all levels of care were invited to participate in the survey electronically. This was achieved by contacting the heads of department at the clinics and requesting that they disseminate the web link within their respective departments. A total of approximately 800 anesthetists were invited to participate in the survey via email. This number includes the members of the airway management working group. In the second part of the survey, 109 anesthetists from the scientific working group on pediatric anesthesia were invited. The detailed response rates are presented in the results. No reminder email was sent. Only complete questionnaires were included in the final analysis.

### Questionnaire development

A questionnaire comprising 18 items was developed for the purpose of analysing the practice of ApOx in anesthesiology. To enhance the quality of the instrument, the items were subjected to a modified cognitive test procedure with the objective of optimising them. The initial questionnaire was evaluated through semi-structured interviews with 25 physicians representing diverse educational backgrounds. Furthermore, the questionnaire was subjected to an expert review by a specialist in medical education. Following the incorporation of the questionnaire into the online platform (LimeSurvey, Hamburg, Germany), the questions were subjected to a second round of review by a panel of 25 medical professionals. The questionnaire was newly developed for this study and has not been published elsewhere. The detailed questions can be found in the supplementary files.

### Inclusion and exclusion criteria

**Fig. 2:**
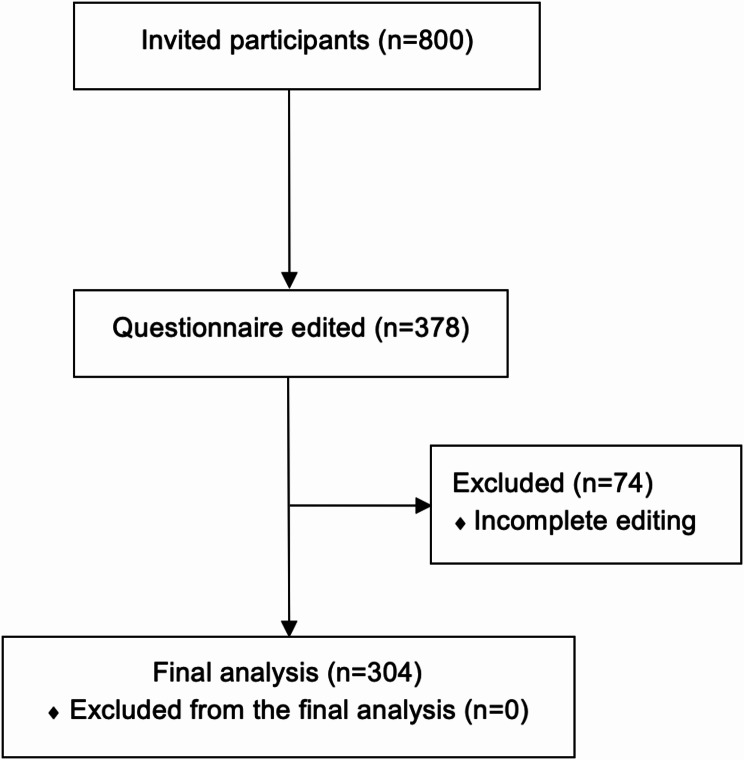
Flowchart of general participant recruitment showing numbers of invited, included, and excluded participants based on the study criteria

The inclusion criteria were as follows: participants must be over the age of 18 years and must be either a physician in further training or a specialist in anesthesiology. The exclusion criteria were as follows: refusal by the participants. After excluding incomplete surveys, a total of 304 participants were included in the final analysis, as shown in Fig. 2.

### Ethics approval and consent to participate

Ethical approval for this study was deemed unnecessary by the local ethics committee of the Medical Faculty Heidelberg, University Heidelberg, Germany due to the absence of identifiable data. Correspondence on 07.06.2024 (§ 15 Abs. 1 BOÄ BW).

After voluntarily confirming the data protection provisions of the survey, participants were able to start answering the questions immediately. Informed consent to participate was obtained from all of the participants before starting the survey. The survey was voluntary, anonymous and could be interrupted by the participants at any time. The participants actively agreed before the survey that the anonymous data may be used and that the data protection regulations will be observed.

### Statistical analysis

The data were collated using Microsoft Excel 2021^®^ (Microsoft, USA) data sheets and subsequently analysed using the statistical software SPSS^®^ version 26.0 (IBM, USA). The results were presented in both absolute numbers and as a percentage of respondents. For continuous data and scores, the mean, standard deviation, minimum, median, and maximum were calculated A comprehensive set of descriptive statistics is provided for all data collected.

## Results

### Baseline and demographic data

Approximately 800 participants were invited to complete the survey via email. Of these, 304 (response rate 38%) edited the survey within a one-month period. Figure 2 illustrates the flowchart of general participant recruitment. The majority of the 304 anesthetists were male 188 (61.8%), with a mean age of 41 years (range: 26–66 years). Table 1 displays the baseline characteristics of the surveyed physicians. Most of the physicians came from university hospitals 105 (34.5%) followed by maximum care hospitals 76 (25.0%), details are shown in Table 1. 201 (66.1%) of the anesthetists surveyed stated that they worked in pediatric anesthesia.

**Table 1:** Baseline characteristics of participating anesthetists. Percentages May not add up exactly to 100% due to rounding effects

Characteristics	No. of Participants (%)
Gender	
Female	106 (34.8%)
Male	188 (61.8%)
Agender	1 (0.3%)
Not specified	9 (3.2%)
**Age (mean)**	41 years (range: 26–66 years)
**Professional Position**	
Resident	101 (33.2%)
Consultant	101 (33.2%)
Senior Physician	76 (25.1%)
Head of Department	23 (7.5%)
**Type of Hospital**	
University Hospital	105 (34.5%)
Maximum Care Hospital	76 (25.0%)
Major Regional Hospital	55 (18.0%)
General Hospital	44 (14.4%)
Not specified	22 (7.3%)
Private Practice	2 (0.7%)
**Additional Specialisation**	
Prehospital Emergency Physician	179 (58.8%)
Intensive Care Medicine	144 (47.3%)

### Process indicators

**Fig. 3:**
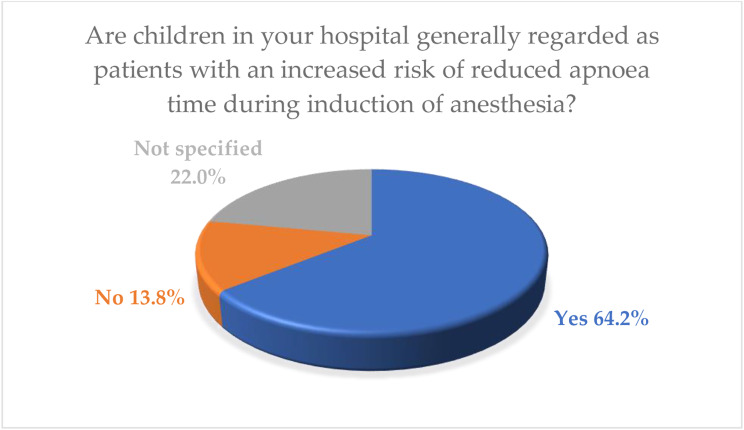
Answer to the question: Are children in your hospital generally regarded as patients with an increased risk of a shortened apnoea time during induction of anesthesia?

Of the respondents 195 (64.2%) considered pediatric patients to be at an increased risk of reduced apnoea time. Conversely, 42 (13.8%) respondents found that children do not have an increased risk in terms of reduced apnoea time. Details are shown in Fig. 3.

**Fig. 4:**
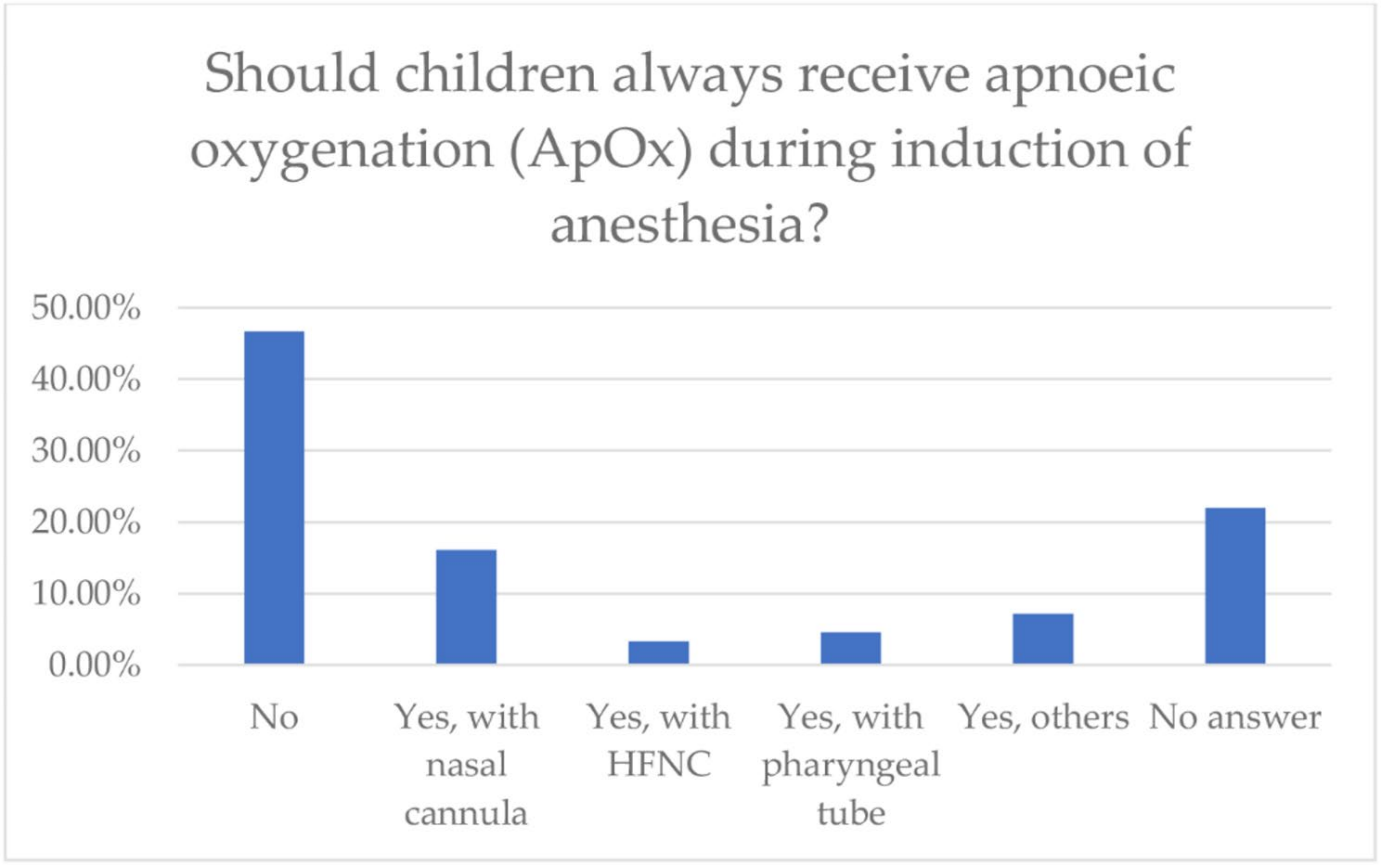
The figure shows the evaluations of whether children should generally have apnoeic ox-ygenation during induction of anesthesia, and if so, by which method. Figures in the bar chart are shown in percent (%). HFNC = High-Flow-Nasal-Cannula

A large number of participants were of the opinion that children should not have always ApOx during induction of anesthesia. The exact details are shown in Fig. 4.

According to 192 (63.2%) of the participants, ApOx in pediatric anesthesia is currently not a standard procedure in their hospitals. Details are shown in Table 2. The use of high-flow nasal cannula (HFNC) with varying flow rates and oxygen concentrations is less prevalent than that of the standard nasal cannula and pharyngeal tube. (HFNC 7.6% vs. 68.1% low-flow nasal cannula/pharyngeal tube).

**Table 2:** This table displays the distribution of answers to the corresponding. RSI = Rapid sequence intubation, HFNC = High-Flow-Nasal-Cannula. Percentages May not add up exactly to 100% due to rounding effects

Question	Answer; *n* (%)
Is apnoeic oxygenation a standard procedure for induction of pediatric anesthesia in your clinic?	No; 192 (63.2%)
	Yes, in neonates; 5 (1.6%)
	Yes, < 1 years; 15 (4.9%) Yes, < 2 years; 3 (0.9%) Yes, < 3 years; 1 (0.3%) Yes, < 4 years; 2 (0.6%) Yes, < 5 years; 2 (0.6%) Yes, < 6 years; 2 (0.6%) Yes, > 6 years; 0 (0.0%) Yes, during RSI; 8 (2.6%) Others; 7 (2.3%) No answer; 67 (22.0%)
If you use apnoeic oxygenation, what is the practical implementation of apnoeic oxygenation in children in your working environment?	Nasal Cannula; 60 (19.7%) HFNC; 24 (7.9%) Pharyngeal tube; 32(10.5%) Others; 22 (7.2%)
	No answer; 67 (22.0%)
If you are using apnoeic oxygenation, what flow rate do you choose for oxygen delivery during apnoeic oxygenation in pediatric patients *over 1 year of age?*	0.2 l/kg/min via nasal cannula/pharyngeal tube; 33 (10.9%)
	1 l/kg/min via nasal cannula/pharyngeal tube; 29 (9.5%)
	8 l/min via nasal cannula/pharyngeal tube; 38 (12.5%)
	HFNC < 20 l/min, FiO2 1.0; 10 (3.3%)
	HFNC > 20 l/min, FiO2 1.0; 9 (2.6%) HFNC < 20 l/min, FiO2 0.3; 1 (0.3%) HFNC > 20 l/min, FiO2 0.3; 0 (0.0%) Others; 96 (31.5%) No answer; 71 (23.4%)
If you are using apnoeic oxygenation, what flow rate do you choose for oxygen delivery during apnoeic oxygenation in pediatric patients *under 1 year of age?*	0.2 l/kg/min via nasal cannula/pharyngeal tube; 59 (19.4%) 1 l/kg/min via nasal cannula/pharyngeal tube; 33 (10.8%) ≤ 2 l/kg/min via nasal cannula/pharyngeal tube; 75 (24.7%) ≥ 6 l/kg/min via nasal cannula/pharyngeal tube; 40 (13.2%)
	HFNC < 10 l/min, FiO2 1.0; 10 (3.3%) HFNC > 10 l/min, FiO2 1.0; 9 (2.9%) HFNC < 10 l/min, FiO2 0.3; 5 (1.4%) HFNC > 10 l/min, FiO2 0.3; 1 (0.3%) Others; 5 (1.6%) No answer; 67 (22.0%)

**Fig. 5:**
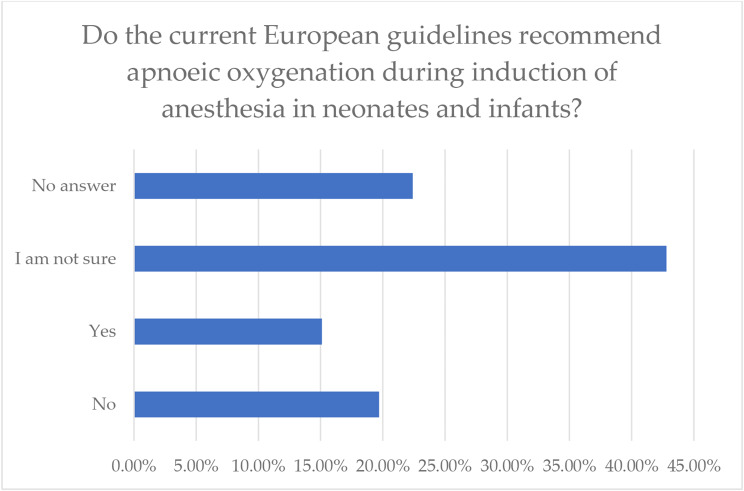
The figure illustrates the extent to which anesthetists are conversant with the currently valid European guidelines for the use of apnoeic oxygenation in neonates and infants

A total of 190 (62.5%) of the participating physicians were unaware that the current European guidelines recommend the use of ApOx in neonates and infants. Details are shown in Fig. 5.

### Complications of apnoeic oxygenation

163 (54.0%) of the participating physicians indicated that they do not anticipate any significant complications when performing ApOx for anesthesia induction. A total of 22 (7.2%) respondents indicated that they were concerned about the potential for complications. A total of 51 (16.8%) respondents indicated uncertainty regarding the potential for complications associated with ApOx. Details are shown in Table 3.

**Table 3:** This table shows the distribution of the answers to the corresponding questions. Percent-ages May not add up exactly to 100% due to rounding effects

Question	Answer; *n* (%)
Are you concerned about the potential for complications associated with this method when performing apnoeic oxygenation during anesthesia induction?	No; 163 (54.0%)
	Yes; 22 (7.2%)
	I am not sure; 51 (16.8%) No answer; 68 (22.7%)
In your opinion, what kind of complications are relevant when performing apnoeic oxygenation?	Hypoxia; 45 (14.8%) Hypercapnia; 47 (15.5%) Risk of aspiration; 22 (7.2%) Barotrauma; 34 (11.2%)
	Mucous membrane irritation; 56 (18.4%) None; 106 (34.9%) Others; 15 (4.9%) No answer; 67 (22.0%)
Other subjective problems/complications mentioned by the participants due to the use of apnoeic oxygenation.	Difficult mask ventilation Prolonged intubation time
	Technical problems Oxygen toxicity in premature infants Stomach distension

### Use of apnoeic oxygenation

A total of 121 (39.8%) respondents indicated that they had never employed ApOx as part of pediatric anesthesia. A further 75 (24.7%) respondents indicated that they seldom utilise this technique. The survey revealed that 17 (5.6%) physicians regularly employ ApOx in pediatric anesthesia. A further 23 (7.6%) respondents indicated that they perform ApOx on an occasional basis. Sixty-six respondents (22.1%) did not provide a response to this question.

### Results of the scientific pediatric anesthesia working group

One hundred and nine members of the scientific working group were invited to participate in the survey, of which 36 completed the entire survey (survey response rate 33%). As illustrated in Table 4, the baseline characteristics of the members of the scientific pediatric anesthesia working group of the DGAI are presented.

**Table 4:** Baseline characteristics of the anesthetists from the pediatric anesthesia working group of the German society of anesthesiology and intensive care medicine (DGAI). Percentages May not add up exactly to 100% due to rounding effects

Characteristics	No. of Participants (%)
Gender	
Female	21 (58.3%)
Male	14 (38.9%)
Agender	0 (0.0%)
Not specified	1 (2.8%)
**Age (mean)**	48 years (range: 34–68 years)
**Professional Position**	
Resident	2 (5.6%)
Consultant	7 (19.4%)
Senior Physician	21 (58.3%)
Head of Department	5 (13.9%)
Not specified	1 (2.8%)
**Type of Hospital**	
University Hospital	21 (58.3%)
Maximum Care Hospital	6 (16.7%)
Major Regional Hospital	5 (13.8%)
General Hospital	0 (0.0%)
Not specified	3 (8.4%)
Private Practice	1 (2.8%)
**Additional Specialisation**	
Prehospital Emergency Physician	32 (88.9%)
Intensive Care Medicine	21 (58.3%)
**Regular work in pediatric anesthesia**	
Yes	34 (94.4%)
No	1 (2.8%)
Not specified	1 (2.8%)

The results of the expert group show that 20 (55.6%) of the pediatric anesthesia experts use ApOx as part of their standard induction procedure for pediatric patients. The question of whether the current European guidelines could recommend ApOx during induction of anesthesia in newborn and infant could be answered correctly at 22 (61.1%). Furthermore, 4 (11.1%) of the experts expressed the opinion that the currently valid guidelines make no recommendations regarding ApOx in newborns and infants. A total of 10 respondents (27.8%) from the working group pediatric anesthesia expressed uncertainty regarding this question. The majority of participants, 23 (63.9%) used low-flow ApOx via a standard nasal cannula, details are shown in Table 5.

**Table 5:** This table shows the distribution of responses to the corresponding question from members of the pediatric anesthesia working group. Percentages May not add up exactly to 100% due to rounding effects

Question	Answer; *n* (%)
If you use apnoeic oxygenation, what is the practical implementation of apnoeic oxygenation in children in your working environment?	Nasal Cannula; 23 (63.9%) HFNC; 4 (11.1%) Pharyngeal tube; 5 (13.9%) Others; 4 (11.1%)
If you are using apnoeic oxygenation, what flow rate do you choose for oxygen delivery during apnoeic oxygenation in pediatric patients *over 1 year of age?*	0.2 l/kg/min via nasal cannula/pharyngeal tube; 11 (30.6%)
	1 l/kg/min via nasal cannula/pharyngeal tube; 8 (22.2%)
	2 l/min via nasal cannula/pharyngeal tube; 5 (13.9%)
	HFNC < 20 l/min, FiO2 1.0; 1 (2.8%)
	Others; 3 (8.3%) No answer; 8 (22.2%)
If you are using apnoeic oxygenation, what flow rate do you choose for oxygen delivery during apnoeic oxygenation in pediatric patients *under 1 year of age?*	0.2 l/kg/min via nasal cannula/pharyngeal tube; 11 (30.6%) 1 l/kg/min via nasal cannula/pharyngeal tube; 10 (27.8%) > 2 l/kg/min via nasal cannula/pharyngeal tube; 3 (8.3%)
	HFNC < 10 l/min, FiO2 1.0; 2 (5,6%) HFNC < 10 l/min, FiO2 0.3; 1 (2.8%) Others; 2 (1.6%) No answer; 7 (19.4%)

In reference to the anticipated complications associated with the utilisation of ApOx, the expert group expressed no major concerns regarding its use. Concerns regarding the occurrence of hypoxia were articulated by 7 (19.4%) of the respondents, despite the utilisation of ApOx. Hypercapnia was a concern for 3 respondents (8.3%), barotrauma for 4 (11.1%), and mucosal irritation for 6 (16.7%).

## Discussion

To the best of our knowledge, this is the first study to examine the utilisation of ApOx in pediatric anesthesia across diverse levels of experts and hospital care. A large proportion of the anesthetists who participated in the survey were unaware that the guidelines on airway management in neonates and infants, which were e-published in December 2023, recommend the use of ApOx. Moreover, there is an absence of established standards in Germany for the utilisation of ApOx, including parameters such as flow rate and the selected method.

### Structure and process indicators

It is not new that ApOx can prolong the safe apnea time without desaturation as part of advanced airway management to increase patient safety, particularly in neonates and infants. Nevertheless, despite the recommendations set forth in the authoritative guidelines, the practice was observed to be employed infrequently in the hospitals under review. This finding is also consistent with the use of ApOx, as evidenced by the survey results, which indicate that only a third of respondents performed ApOx in different ways. This proportion was higher in the group of respondents from the scientific pediatric anesthesia working group, where just over half of the respondents used ApOx. Nevertheless, the remaining experts in pediatric anesthesia in Germany have not yet adopted ApOx as a standard procedure in their departments. In our survey, a large number of questions pertaining to ApOx in children were left unanswered. This is likely attributable to the fact that the majority of anesthetists do not have routine contact, particularly with young children undergoing anesthesia. Despite the fact that approximately two-thirds of the study’s participants indicated that they work in pediatric anesthesia, it is not possible to provide a comprehensive analysis in this respect. This is due to the fact that the participants were not asked to provide detailed information regarding the age range of the children treated, and the frequency of working in pediatric anesthesia.

In its current guidelines, the European Society of Anesthesiology and Intensive Care (ESAIC) recommends the use of ApOx in advanced airway management of neonates and infants [18]. The recommendations include the use of ApOx during tracheal intubation in newborns (level of recommendation: 1B), in infants, the use of apnoeic oxygenation should be based on the risk of hypoxemia in the patient and the experience of the provider (level of recommendation: clinical practice statement) [18]. In response to the question of whether ApOx is recommended as standard for neonates and infants in the European guidelines, a considerable degree of uncertainty was evident in our survey. Only 15.1% of the participants in our survey showed awareness of ApOx recommendation for neonates and infants. In the selected group of anesthetists from the DGAI working group on pediatric anesthesia, more than 60% of the respondents were familiar with the recommendations for ApOx. However, more than a third of the experts also showed uncertainty. There were no major differences between the various hospital- and education levels of the physicians. It appears that the dissemination of current guidelines is not as widely known at the medical level as might be expected. However, it is evident from earlier surveys that recommendations or methods require a certain period of time before they are widely adopted in practice [15, 16]. It is important to acknowledge that the dissemination of a guideline within a community may take some time following its publication. However, the reasons for the lack of adherence to the guidelines are beyond the scope of this study and therefore cannot be investigated. In general, the available data on adherence to and implementation of airway management guidelines is limited [19].

### Goals and effects of apnoeic oxygenation

It is our contention that the infrequent use of ApOx is not a reasonable approach, given that the method has the potential to significantly extend the safe apnoea time without desaturation and have positive effects on the first pass success [2, 12, 14, 20, 21]. It is thus our intention to draw attention to a number of factors with a view to enhancing the efficacy of the ApOx:

In a randomised controlled trial, which assessed apnoea times during intubation of children with the use of low flow oxygen applied via a nasal cannula attached to the laryngoscope and found longer apnoea times compared to laryngoscopy without oxygen insufflation [22]. These differences were confirmed in randomised controlled high-quality studies, regardless of whether a low-flow nasal cannula or high-flow nasal cannula (THRIVE) was used for ApOx [12]. The low-flow nasal cannula can be administered with minimal effort via a standard nasal cannula through the existing oxygen connection port. Consequently, this intervention does not necessitate the use of additional devices, and the effort and resistance required by staff could be reduced when using the standard nasal cannula in comparison to THRIVE [2, 14]. This is due to the fact that these high-flow systems typically necessitate the use of a standalone device and are not universally accessible at the anesthesia workstation. Given the heterogeneity of the available scientific studies, it is not possible to determine with certainty which method (high-flow vs. low-flow) should be preferred [2, 14, 18].

In the HAMSTER Trial published this year, which compared high-flow during tubeless upper airway surgery with standard care (low-flow), no benefit was achieved by using high-flow [23]. The rate of adverse events between the groups also showed no differences. Further studies are required to establish whether high-flow and low-flow are equally effective in prolonging safe apnoea time without desaturation, or whether high-flow is superior.

In pediatric advanced airway management, the number of tracheal intubation attempts is a critical factor, as the incidence of adverse events is directly associated with the overall number of attempts [20, 24, 25]. A recent meta-analysis of ApOx by Fuchs et al. indicates that ApOx reduces the overall number of tracheal intubation attempts by enhancing the initial success rate [2]. This is of particular importance for neonates, infants of a smaller stature and children with limited cardiopulmonary reserve, as they are more susceptible to rapid oxygen desaturation. Patients in this age group often exhibit elevated oxygen consumption, diminished closing capacity, reduced functional residual capacity, and an elevated risk of airway collapse relative to older children [2, 26]. In conclusion, the evidence indicates that ApOx can facilitate a secure time window without desaturation for advanced airway management in pediatric patients and it can increase patient safety during general anesthesia in children. Furthermore, the use of a standard nasal cannula is well known, widely available and inexpensive.

### Complications of apnoeic oxygenation

According to our survey results, there is a relatively low level of concern about potential adverse events associated with ApOx. Given that the physicians surveyed had undergone extensive training (65.8% were consultants, senior physicians, or heads of departments), this assessment is likely to be valid. Although the evidence for the negative effects of ApOx is limited, there is no evidence that apnoeic oxygenation is harmful [14]. A pediatric study using the high-flow nasal cannula as part of ApOx found no evidence of gastric insufflation or pneumothorax using ultrasound [12]. It is important to note that the study had a relatively small sample size, which may have affected the reliability of the results. Additionally, other scientific studies indicate that there is no discernible evidence of gastric bloating when ApOx is employed [27, 28]. One assertion that is repeatedly made in the context of ApOx is the development of oxidative stress due to the high oxygen concentrations. It should be noted that there is currently no available scientific data on this topic in children. As with all such cases, the information must be subjected to critical analysis, as hypoxia is also a potentially fatal condition that can result in bradycardia and cardiac arrest [2]. Nevertheless, the treating medical team should critically assess the use of ApOx in children with relevant pre-existing conditions, such as cyanotic congenital heart disease.

### Technical conditions

As previously discussed, various methodologies and oxygen flow rates are available for ApOx. However, this survey found no standardization in either group regarding method or oxygen dosage. Further research is needed to establish appropriate flow rates and develop age- or weight-adjusted recommendations. Some respondents to the survey indicated that the technical prerequisites for conducting an ApOx under anesthesia were not fully satisfied in all operating theatres. To illustrate, some locations lack the supplementary oxygen port that is essential for nasal cannulas. It is our considered opinion that this aspect should be subjected to the most rigorous scrutiny. The field of anesthesia is inherently high-risk, and it is of the utmost importance to take every possible measure to enhance patient safety, particularly in settings where pediatric patients are treated. The majority of hospitals utilise a central gas/air system, which should facilitate the establishment of an additional oxygen connection-port, particularly in the aforementioned areas where pediatric anesthesia is administered. In the event that this is not technically feasible, a mobile oxygen cylinder can be employed for anesthesia induction. It is therefore recommended that this aspect of the technical requirements be included in the guidelines as a fundamental prerequisite to enhance patient safety.

### Suggestions

Participants were also able to write down their wishes or suggestions as part of our survey. One concern raised by the anesthetists interviewed regarding the use of ApOx was the potential for difficult mask ventilation due to the nasal cannula. This phenomenon can also be replicated in our clinical experience. As a result, our approach is to use the nasal cannula only after the end of mask ventilation and shortly before the start of tracheal intubation. It is important to note that the aforementioned suggestion does not take into account the issue of a failed first intubation attempt, which necessitates subsequent mask ventilation. At this juncture, it is also imperative to consider the necessity to maintain an adequate depth of anesthesia during airway management and the potential for volatile anesthetic agent washout, which can result in both a reduced depth of anesthesia and contamination of the operating environment. It is acknowledged that a certain latency period exists between the publication of guideline recommendations and their practical implementation. In recent years, medical education has been characterised by various online platforms and blogs in addition to scientific publications. This development has undoubtedly created a valuable opportunity to raise users’ awareness of new recommendations and to facilitate more rapid implementation through the publication of additional online material following the dissemination of a guideline. This dissemination could be facilitated by medical associations, for instance, through the dissemination of newsletters or analogous formats to their members across various geographical regions.

Furthermore, a large number of participants described their positive experiences with ApOx and considered it important to continue promoting this topic.

### Limitation

The response rate for the survey was 38%, which is a satisfactory result in comparison to similar surveys [29, 30]. This is particularly pertinent given that the participants in our survey were not offered any form of remuneration [31]. We surveyed a general cohort of anesthetists and not just those who are experts in pediatric anesthesia. Although many of the respondents were working in pediatric anesthesia, this could also have an influence on the results. Following the publication of medical guidelines, a period of time is typically required for them to become established. It is therefore possible that the timing of the survey may also have an impact on the interpretation of the results, despite the fact that the ApOx is not a novel method. It is possible that the limited duration of the survey and the time required by participants may have constituted obstacles to higher levels of participation. Furthermore, it was a single survey without a follow-up. The survey was conducted during the summer period, which might have influenced the response rate. The anonymity of the online survey means that social desirability is a less significant factor, making it a superior format to other survey types [32]. The strength of the present survey lies in the targeted selection of respondents, which ensures a homogeneous sample of participants relevant to the topic under investigation [33, 34].

## Conclusions

Notwithstanding the recommendations set forth in the existing European guidelines, the use of ApOx does not appear to be a standard practice in Germany. While the reasons for this remain unclear, they should be addressed in future scientific studies.

Furthermore, the anesthetists interviewed expressed considerable uncertainty regarding the use of ApOx, even in the scientific working group of pediatric anesthesia. There seems to be no standardisation with regard to the implementation of ApOx, including aspects such as the type and oxygen flow rates. Further enhancements are required to improve patient safety in the context of pediatric advanced airway management. In our view, ApOx should become a standard procedure in pediatric anesthesia, especially for neonates, infants and critically ill children.

## Electronic supplementary material

Below is the link to the electronic supplementary material.

Supplementary Material 1

## Data Availability

The authors confirm that the data supporting the findings of this study are available within the article. The datasets used during the current study are available from the corresponding author on reasonable request.

## Abbreviations

ApOx: Apnoeic Oxygenation
DGAI: German Society of Anesthesiology and Intensive Care Medicine
FIO2: Fractional inspired oxygen (FIO2)
HFNC: High-flow nasal cannula
PEEP: Positive end-expiratory pressure
RSI: Rapid Sequence Intubation
THRIVE: Transnasal Humidified Rapid Insufflation Ventilatory Exchange
