# Supplementary material for: Apnoeic oxygenation in pediatric anesthesia: better safe than sorry!
Source: BMC Anesthesiol. 2025 Mar 8;25:116. doi: 10.1186/s12871-025-02995-2 (PMC11889762; doi:10.1186/s12871-025-02995-2)
Supplement: Supplementary file 1 — Supplementary Material 1 [file 12871_2025_2995_MOESM1_ESM.pdf]

Apneic oxygenation in pediatric anesthesia

Dear Colleagues,

Welcome to our online survey **"Apneic oxygenation in pediatric anesthesia".**

With your participation, you will make a valuable contribution to outlining the standard of anesthesia induction in high-risk patients and identifying approaches to improve and optimize medical care.

Answering the questionnaire will take about 5 minutes of your time. The online survey is completely anonymous and voluntary. The Ethics Committee of the Medical Faculty of Heidelberg University has not expressed any concerns about this survey.

Thank you in advance for your support and your time.

For further information or queries, please contact our colleagues at the Department of Anesthesiology at Heidelberg University Hospital at any time.

Contact person for the survey:

Dr. med. D. D. Uzun

Deniz.Uzun@med.uni-heidelberg.de

Physician of the Clinic for Anesthesiology (Medical Director Prof. Dr. med. M. A. Weigand)

Heidelberg University Hospital

69120 Heidelberg

and

PD Dr. med. F. Schmitt, MHBA, DESAIC

Deputy Medical Director of the Clinic for Anesthesiology (Medical Director Prof. Dr. med. M. A. Weigand)

Heidelberg University Hospital

69120 Heidelberg

* For better readability, the generic masculine is used in this survey. Unless otherwise indicated, the personal designations used in this work refer to all genders.

This survey contains 18 questions.

Apneic oxygenation in pediatric anesthesia

Please enter your age:

*

Only numbers may be entered in this field.
Please enter your answer here:

-

What applies to you?

*

Please select the answers that apply:
Please select all applicable answers:

- Female
- Male
- Miscellaneous
- Not specified

What level of training do you have?

*

Please choose one of the following answers:
Please select only one of the following answers:

- Doctor in further training
- Specialist
- Senior physician
- Chief physician

Into which supply level can your main workplace be divided?

*

Please choose one of the following answers:
Please select only one of the following answers:

- Basic and standard care providers
- Maximum provider
- University Hospital
- Main provider
- Outpatient activity
- Miscellaneous

Do you have the additional qualification in emergency medicine ?

Please select only one of the following answers:

- Yes
- No

Do you have an additional qualification in intensive care medicine ?

Please select only one of the following answers:

- Yes
- No

Do you regularly work in pediatric anesthesia ?

Please select only one of the following answers:

- Yes
- No

Does your hospital have a standard of care (SOP) with structured instructions for apneic oxygenation during induction of anesthesia?

*

Please choose one of the following answers:
Please select only one of the following answers:

- No
- Yes (clinic operating area)
- Yes (hospital intensive care unit)
- Yes (emergency medical service)

Are children in your hospital generally regarded as patients with an increased risk of reduced apnea time during induction of anesthesia?

*

Please select only one of the following answers:

- Yes
- No

Is apneic oxygenation a standard procedure for the induction of pediatric anesthesia in your clinic?

*

Please select the answers that apply:
Please select all applicable answers:

- No
- Yes (newborns only)
- Yes (children <1 year of age)
- Yes (children <2 years of age)
- Yes (children <3 years of age)
- Yes (children <4 years of age)
- Yes (children <5 years of age)
- Yes (children >5 years old)
- Yes (as part of an RSI)
- Other:

In your opinion, do the current guidelines recommend standard apneic oxygenation during induction of anesthesia in newborns and infants?

*

Please choose one of the following answers:
Please select only one of the following answers:

- Yes
- No
- I am not sure

How does the practical implementation of apneic oxygenation in children work in your working environment?

*

Please select the answers that apply:
Please select all applicable answers:

- Standard nasal cannula
- High-flow nasal cannula (HFNC)
- Vengeance tube
- I do not perform apneic oxygenation
- Other:

What flow rate do you choose for oxygen delivery for apneic oxygenation in pediatric patients over 1 year of age?

*

Please select the answers that apply:
Please select all applicable answers:

- 0.2l/kg/min via nasal cannula/reach tube
- 0.4l/kg/min via nasal cannula/reach tube
- 1l/kg/min via nasal cannula/reach tube
- 2l/kg/min via nasal cannula/reach tube
- High-flow nasal cannula <20l/min, FiO2 1.0
- High-flow nasal cannula >20/min, FiO2 1.0
- High-flow nasal cannula <20/min, FiO2 0.5
- High-flow nasal cannula >20/min, FiO2 0.5
- Other:

What flow rate do you choose for oxygen delivery during apneic oxygenation in pediatric patients under 1 year of age?

*

Please select the answers that apply:
Please select all applicable answers:

- 0.2l/kg/min via nasal cannula/reach tube
- 0.4l/kg/min via nasal cannula/reach tube
- 1l/kg/min via nasal cannula/revenge tube
- <2l/min via nasal cannula/reach tube
- >2l/min via nasal cannula/reach tube
- High-flow nasal cannula <10l/min, FiO2 1.0
- High-flow nasal cannula >10/min, FiO2 1.0
- High-flow nasal cannula <10/min, FiO2 0.5
- High-flow nasal cannula >10/min, FiO2 0.5
- Other:

Are you worried about complications that could be caused by this method when performing apneic oxygenation during induction of anesthesia?

*

Please select the answers that apply:
Please select all applicable answers:

- Yes
- No

- I am not sure

What kind of complications do you think are relevant when performing apneic oxygenation?

*

Please select the answers that apply:
Please select all applicable answers:

- None
- Hypoxia: Insufficient oxygen supply despite the method
- Hypercapnia: increase in the carbon dioxide content in the blood, as CO2 removal is not guaranteed
- Aspiration hazard
- Barotrauma: injuries caused by high pressure in the airways
- Mucous membrane irritation: Dry or irritated mucous membranes due to the oxygen supply
- Other:

How often have you personally performed apneic oxygenation in children?

*

Please select the answers that apply:
Please select all applicable answers:

- Never before
- Very rare
- Rare
- Occasionally
- Regular (standard)
- Other:

Do you have any further comments on the topic of apneic oxygenation in pediatric anesthesia?

Please enter your answer here:

Submission of your completed questionnaire:
Thank you for answering the questionnaire.
